# Comparative performance of aldolase and lactate dehydrogenase rapid diagnostic tests in *Plasmodium vivax* detection

**DOI:** 10.1186/1475-2875-13-272

**Published:** 2014-07-11

**Authors:** Emmanuel E Dzakah, Keren Kang, Chao Ni, Shixing Tang, Jihua Wang, Jufang Wang

**Affiliations:** 1 School of Bioscience and Bioengineering, South China University of Technology, University City, Panyu District, Guangzhou 510006, China; 2 Department of Molecular Biology and Biotechnology, School of Biological Sciences, University of Cape Coast, Cape Coast, Ghana; 3 National Engineering Laboratory of Rapid Diagnostic Tests, Guangzhou Wondfo Biotech Co Ltd, Science City, Lizhishan Rd No 8, Luogang District, Guangzhou 510663, China

**Keywords:** *Plasmodium vivax*, Antigen, Aldolase, Misdiagnosis, Malaria

## Abstract

**Background:**

Misdiagnosis of malaria by commercial rapid diagnostic tests (RDTs) is a major cause of concern in the diagnosis of malaria. This retrospective study was aimed at assessing the relative performance of four RDTs with emphasis on the detection of two *Plasmodium vivax* antigens: aldolase and lactate dehydrogenase (LDH).

**Methods:**

Three commercially available *Plasmodium* LDH or aldolase antigen detection kits (One Step Malaria P.f/P.v, ParaHit Total ver. 1.0, SD Bioline Malaria) and an anti-*P. vivax* aldolase-specific monoclonal antibody (mAb) pair 1C3-12 F10 were evaluated with *P. vivax* positive as well as non-*P. vivax* samples and healthy samples using blood smear examination as standard. Each test was read according to the manufacturer’s instructions.

**Results:**

MAb 1C3-12 F10 pair targeting *P. vivax*-specific aldolase exhibited very good specificity and sensitivity of 100 and 97.4%, respectively. Positive predictive value (PPV) and negative predictive value (NPV) of 100 and 99.5%, respectively, were also observed. The anti-*P. vivax* LDH in the One-Step Malaria P.f/P.v test showed sensitivity, specificity, PPV and NPV of 93.5, 98.0, 88.9 and 98.8%, respectively. ParaHit Total ver. 1.0 targeting the pan-aldolase antigen showed sensitivity, specificity of 97.4 and 99.6%, respectively. PPV and NPV were both 99.5%. SD Bioline had sensitivity, specificity, PPV and NPV of 93.5, 100, 100 and 98.8%, respectively. The overall sensitivity and specificity of all four RDTs were acceptable, especially for the aldolase detection tests. Five (6.5%) of the *P. vivax*-positive samples (n = 77) that were confirmed by microscopic examination as well as the two aldolase detection RDTs (mAb 1C3-12 F10 and ParaHit Total ver.1.0) were undetected by the two LDH detection RDTs (One Step Malaria P.f/P.v and SD Bioline). Similarly, two positive samples (2.6%) that were positively confirmed by the LDH detection RDTs were also undetected by the aldolase detection test kits.

**Conclusion:**

Aldolase and LDH antigens perform differently in different *P. vivax* samples; hence there is a high risk of misdiagnosis when monoclonal antibodies are used against only one particular antigen in the test. A combination of both aldolase and LDH in RDTs for the rapid diagnosis of *P. vivax* will enhance the sensitivity of the assay and reduce misdiagnosis.

## Background

Malaria is a deadly infectious disease with a global impact extending from the most developed countries to the most remote regions of the world [1]. Worldwide malaria mortality rates have been significantly reduced over the last decade by 45% in all age groups and by 51% in children under five years of age [2]. This is a significant success and highlights progress towards the global malaria targets of reducing the incidence rate by 75% by 2015 [3]. However, there is no need for complacency but rather a more focused approach to ensure that misdiagnosis which results in treatment delays [4] and subsequent mortality does not occur in future.

The standard method of malaria diagnosis is by the routine microscopic examination of Giemsa-stained blood smears [5]. Fluorescent staining methods such as quantitative buffy coat (QBC) have also been recommended for use in malaria diagnosis [6]. The emergence of rapid test assays for easy and inexpensive diagnosis of malaria infection has been effective in areas where expertise in microscopic diagnosis is unavailable [7]. Rapid diagnostic tests (RDTs) that detect malaria parasite proteins by immunochromatography have been used as complementary detection method for malaria diagnosis [8,9]. These tests are convenient and simple to operate and facilitate the rapid testing of clinical specimens within 10 to 30 minutes [10]. RDTs for malaria offer the greatest possibility of extending accurate malaria diagnosis to remote areas where trained personnel, microscopes and other equipment are not easily accessible [11]. Malaria RDTs have been developed with great focus on the detection of histidine-rich protein 2 (PfHRP2) from *Plasmodium falciparum* and parasite-specific lactate dehydrogenase (pLDH) or *Plasmodium* aldolase (pALDO) from all species [7]. The PfHRP2-based test is a reliable complimentary test to routine microscopy for the diagnosis of *P. falciparum* malaria [12], however, the relative sensitivity and specificity of RDTs to non-*P. falciparum* species is low [11-13].

Recent advances in the development of species-specific RDTs, such as the *Plasmodium vivax* aldolase-specific test [14], will greatly enhance the quality of testing and reduce over-administration of anti-malarial drugs in endemic areas to a more species-specific approach to the treatment of malaria.

In this research, the comparative study of the diagnostic performance of four rapid malaria RDTs (One Step Malaria P.f/P.v, ParaHit Total ver. 1.0, SD Bioline Malaria and monoclonal antibody (mAb) pair 1C3-12 F10) is reported. The 1C3-12 F10 mAb pair was selected from a number of anti-aldolase mAb secreting clones, developed to specifically detect only the *P. vivax* aldolase antigen as reported earlier [14]. One Step Malaria P.f/P.v (Guangzhou Wondfo Biotech Co Ltd, China) contains an anti-LDH mAb pair that is specific to *P. vivax* LDH antigen, and SD Bioline Malaria (Standard Diagnostics, Korea) detects the pan-LDH antigen, while the ParaHit Total ver.1.0 (Span Diagnostics Ltd, India) detects the pan-aldolase antigen. The relative performance of these RDTs was compared for their ability to detect the *P. vivax* parasite in clinical samples.

## Methods

### Sample collection

Venous blood sample taken in an anticoagulant (EDTA)-containing tube was used for thick and thin blood smears. Sample tubes were stored at −20°C until needed for laboratory examination. Thick and thin blood smears were prepared and read as described earlier by the WHO standard method [15]. Each slide was independently examined by two experienced microscopists at the Yunnan Provincial Institute of Parasitic Diseases, who were blinded to the patients’ characteristics and symptoms. *Plasmodium vivax*-positive blood samples (n = 77) and non-*P. vivax* samples (n = 33) were collected from patients in the Yunnan Province of China. Non-*P. vivax* samples included *P. falciparum* (n = 31) and *P. malariae* (n = 2) samples. Healthy blood samples (n = 423) were randomly collected from volunteers in Guangzhou. These volunteers had no recent history or symptoms of malaria infection and were diagnosed as having no *Plasmodium* species in the blood.

### Diagnosis with rapid diagnostic tests

Three commercially available *Plasmodium* LDH or aldolase antigen detection kits (One Step Malaria P.f/P.v, ParaHit Total ver. 1.0, SD Bioline Malaria) and an anti-*P. vivax* aldolase mAb pair 1C3-12 F10 were used (Table 1). The test was conducted using anti-coagulated venous blood. Each test was performed and read within a specific time interval according to the manufacturer’s instructions. Test was recorded as positive if both the test (T) band corresponding to either LDH or aldolase, and control (C) band appeared; if only the C line was seen, it was recorded as negative according to the manufacturer’s instruction. Each test was observed by two experienced technicians who were blinded to the results of one another. Band intensity at the end of the stipulated reaction time was compared to a standard colour chart with colour range between C1 (deepest) to C8 (faintest). Negative samples were designated as C9.

**Table 1:** **Detailed information on *Plasmodium vivax *** **rapid diagnostic tests evaluated in this study**

**Test assay**	**Manufacturer/source**	***P. vivax *** **antigen**	**Specimen**	**Format**	**Time**	**Method of analysis**
mAb 1C3-12 F10	Developed by Dzakah *et al*. [14]	*P. vivax* aldolase (PvALDO)	Whole blood	Cassette	15 min	Eye
One Step Malaria P.f/P.v	Guangzhou Wondfo Biotech, China	Pan-LDH (pLDH)	Whole blood	Cassette	15 min	Eye
ParaHit Total ver. 1.0	Span Diagnostics Ltd, India	Pan -aldolase (pALDO)	Whole blood	Cassette	25 min	Eye
SD BIOLINE Malaria	Standard Diagnostics, Korea	Pan-LDH (pLDH)	Whole blood	Cassette	20 min	Eye

### Data analysis

The sensitivity and specificity of the immunochromatographic assay for the detection of *P. vivax* were compared with thick blood microscopic examination results by Kappa statistical analysis, K. P < 0.005 was considered as significant.

## Results

Three commercial RDTs and an antibody pair developed earlier were investigated in this study. The various RDTs were retrospectively studied for their ability to detect either the aldolase or LDH antigen in clinical samples.

All five *P. vivax*-positive samples which showed false negative results in the two LDH-detecting categories were detected with very strong bands in the aldolase-detecting category. Similarly, the two positive samples that were not detected in the aldolase category were however strongly detected in the LDH category (Table 2). Samples showing no bands were graded C9 for respective test kit (Table 3).

**Table 2:** Comparison of four malaria rapid diagnostic tests with microscopic examination

**Assay category**	***P. vivax***-**positive samples**		***P. vivax***-**negative samples**	
	+(%)	-(%)	+(%)	-(%)
mAb 1C3-12 F10	75 (97.4)	2 (2.6)	0 (0)	456 (100)
One Step Malaria P.f/P.v	72 (93.5)	5 (6.5)	9 (2.0)	447 (98.0)
ParaHit Total ver. 1.0	75 (97.4)	2 (2.6)	2 (0.4)	454 (99.6)
SD Bioline Malaria	72 (93.5)	5 (6.5)	0 (0)	423 (100)

*Plasmodium vivax*-negative samples refer to both non-*P. vivax* specimen as well as specimen collected from healthy individuals with no history of malaria infection. In the case of SD Bioline, *P. vivax* negative samples included only healthy samples.

“+ and -” represent the number of detected and undetected specimen, respectively.

**Table 3:** Band **intensity of different target antigen rapid diagnostic tests in the detection of *Plasmodium vivax *** **specimen**

**Test assay**	**Negative (C9)**	**Fair (C6-C8)**	**Moderate (C3-C5)**	**Strong (C1-C2)**	**Total**
mAb 1C3-12 F10	2	12	17	46	77
One step malaria P.f/P.v	5	9	14	49	77
ParaHit total ver. 1.0	2	11	18	46	77
SD bioline malaria	5	7	15	50	77

Band intensities were recorded independently by two individuals who were blinded to the observation of each other.

MAb 1C3-12 F10 pair exhibited excellent specificity and sensitivity of 100% (95% Confidence interval (CI): 99.1-100.0%) and 97.4% (95% CI: 90.9-99.6%), respectively (Table 4). The sensitivity and specificity observed by the One-Step Malaria P.f/P.v test were 93.5% (95% CI: 85.5-97.8%) and 97.9% (95% CI: 96.0-99.0%), respectively. Sensitivity and specificity of ParaHit Total ver. 1.0 were 97.4% (95% CI: 90.9-99.6%) and 99.5% (95% CI: 98.3-99.9%), respectively. SD Bioline had similar sensitivity (93.5%) as observed in One-Step Malaria P.f/Pv but an excellent specificity of 100% (95% CI: 99.1-100.0%). All non-*P. vivax* samples were negative in the 1C3-12 F10 mAb pair combination, OneStep Malaria P.f/P.v and ParaHit Total ver 1.0 test kits as expected since the antigens used in these assays either target the *P. vivax* specific LDH and aldolase or pan-specific aldolase. These were however positive in the SD Bioline assay as the detection antigen was pan-LDH. The general performance of these tests kits were good compared with the microscopy gold standard observation (Table 4).

**Table 4:** **Sensitivity**, **specificity**, **positive predictive value**, **negative predictive value and Kappa comparison of the four rapid diagnostic tests**

**Test assay**	**Sensitivity (%)**	**Specificity (%)**	**PPV (%)**	**NPV (%)**	**Kappa, K**
1C3-12 F10	97.4	100	100	99.5	0.9844
One-step malaria	93.5	98.0	88.9	98.8	0.8958
ParaHit total	97.4	99.6	99.5	99.5	0.9697
SD bioline	93.5	100	100	98.8	0.9609

*PPV*, Positive predictive value; *NPV*, Negative predictive value.

## Discussion

The significance of the use of RDTs in reducing malaria mortality and prevalence cannot be over-emphasized. Nonetheless, misdiagnosis of malaria contributes largely to the over-prescription of anti-malarial drugs such as artemisinin leading to the rise in drug resistance globally [16], with its consequent delay or incomplete clearance of parasites from the patient’s blood [4,16]. *Plasmodium* resistance has been documented in three of the five malaria species known to infect humans: *P. falciparum*, *P. vivax* and *Plasmodium malariae*. Thus, improving the accuracy of malaria diagnosis is becoming more pivotal and must be treated with all the seriousness it deserves in the future.

The present study investigated the performance of malaria RDTs targeting either the *P. vivax*-specific aldolase or LDH antigens or the pan-specific forms compared to the standard microscopic blood smear examination. The anti-aldolase mAb pair 1C3-12 F10 and the anti-LDH mAbs used in the One-Step Malaria P.f/P.v specifically detect only the *P. vivax* while the mAb pairs used in the ParaHit Total ver. 1.0 and SD Bioline are pan-specific aldolase and LDH, respectively, as indicated in Table 1. The tests were performed according to the manufacturer’s instruction and then read at the stipulated time.

Previous studies on the SD Bioline RDT reported sensitivity ranges between 92.7 and 98.8% [17,18] when frozen and fresh samples from South Korea were used. In this study, there was an observed sensitivity of 93.5% and an excellent specificity of 100%. The difference in the values might have appeared as a result of the different samples evaluated and the regions from which they were collected. Earlier study on anti-*P. vivax* aldolase antibodies reported a sensitivity of 98.3% and specificity of 99.2% [14]. The anti-*P. vivax* aldolase mAb pair used in this research estimates improved specificity of 100% for *P. vivax* and a slightly reduced sensitivity 97.4%. One-Step Malaria P.f/P.v, which uses anti-*P. vivax*-specific LDH mAb showed sensitivity and specificity of 93.5% and 98.0%, respectively. The pan-specific aldolase antigen ParaHit Total ver. 1.0 test showed sensitivity of 97.4% and specificity of 99.6% (Table 4). All four RDTs employed in this study showed good agreement with the microscopic smear examination.

While the specificity of the different RDTs varies in the detection of *P. vivax*, one most important finding in this study is the similarity in the percentage sensitivities observed by the different categories of RDTs from different manufacturers but targeting a particular antigen of interest. MAb pair 1C3-12 F10 and ParaHit Total ver.1.0 target the aldolase antigen, although the 1C3-12 F10 mAb pair is *P. vivax*-specific while the ParaHit Total ver. 1.0 is pan-specific in nature. Both tests estimated an overall sensitivity of 97.4%, much higher than the 93.5% observed for the LDH target in the One-Step Malaria P.f/Pv (*P. vivax*-specific LDH) and the SD Bioline (pan-LDH). An interesting observation made in this study was the fact that the two undetected samples in the aldolase category (1C3-12 F10 and ParaHit Total ver. 1.0) were all detected with very strong bands by the LDH category (One-Step Malaria P.f/P.v and SD Bioline). Similarly, all the five undetected *P. vivax* samples in the LDH category were all detected with strong bands by the aldolase category (Table 3). From this result, it is infered that the use of aldolase as a target antigen for the detection of *P. vivax* in clinical samples may provide more reliable diagnosis of the parasite. However, because both aldolase and LDH performed differently in different samples, there is the risk of misdiagnosis when monoclonal antibodies are used against only one particular antigen in the test. A combination of both aldolase and LDH in RDTs for the diagnosis of *P. vivax* will greatly improve the sensitivity of the assay.

## Competing interests

The authors declare that they have no competing interests.

## Authors’ contributions

EED and KK performed the experiments; JW and JW designed the study; CN and ST assisted in data analysis and sample collection. All authors read and approved the final manuscript.
